# Comprehensive analysis and validation of the prognostic significance of cuproptosis-related genes in rectal adenocarcinoma

**DOI:** 10.3389/fcell.2025.1622829

**Published:** 2025-11-27

**Authors:** Tianhao Li, Xinyu Gao, Shuocun Zhang, Haoren Jing, Zhicheng Pu, Xipeng Zhang, Mingqing Zhang

**Affiliations:** 1 Department of colorectal surgery, Tianjin Union Medical Center, The First Affiliated Hospital of Nankai University, Tianjin, China; 2 Tianjin Institute of Coloproctology, Tianjin, China; 3 Department of General Surgery, Tianjin Hongqiao Hospital, Tianjin, China; 4 Nankai University School of Medicine, Nankai University, Tianjin, China

**Keywords:** rectal adenocarcinoma, prognosis, tumor microenvironment, drug sensitivity, risk stratification

## Abstract

**Background:**

Rectal adenocarcinoma has a high incidence and suboptimal therapeutic outcomes. Notably, reliable prognostic biomarkers are currently lacking. Cuproptosis is a newly identified regulated cell death mechanism, and its molecular pathways and implications for rectal adenocarcinoma remain poorly understood. However, compared with colon adenocarcinoma, multi-omics studies of cuproptosis-related genes for rectal adenocarcinoma remain blank.

**Methods:**

This study is the first to construct a cuproptosis-related genes prognostic prediction model for rectal adenocarcinoma by integrating genomics, transcriptomics, and clinical data. Transcriptomic data from the Gene Expression Omnibus and The Cancer Genome Atlas databases were analyzed. Cuproptosis-related genes were curated from prior literature and FerrDb V2. Consensus clustering was employed to identify molecular subtypes based on cuproptosis-related genes expression profiles. Subsequently, functional enrichment and survival analyses were conducted across subtypes. Following univariate Cox regression, LASSO regression, and multivariate analyses, four pivotal cuproptosis-related genes (PPAT, NHP2, INHBB, and MSMP) were selected to construct a prognostic risk model. The associations between risk scores, tumor microenvironment, immune cell infiltration, and drug sensitivity were further investigated.

**Results:**

A total of 30 cuproptosis-related genes were screened, including 16 upregulated and 14 downregulated in rectal adenocarcinoma. These genes were enriched in pathways related to DNA/RNA metabolism, MAPK signaling, and calcium homeostasis. The LASSO regression risk model and nomogram demonstrated robust predictive accuracy for survival outcomes. Notably, cuproptosis-related genes were significantly correlated with immune cell infiltration and checkpoint gene expression, suggesting their dual role in tumor progression and immunomodulation.

**Conclusion:**

The prognostic system based on cuproptosis-related genes constructed in this study not only enhances the accuracy of survival prediction for rectal adenocarcinoma patients, revealing the potential role of cuproptosis in the tumor microenvironment and immune regulation, but also provides a new perspective for risk stratification and the selection of personalized treatment strategies, paving the way for precise oncology approaches in rectal adenocarcinoma.

## Introduction

Colorectal cancer (CRC) is a high-incidence malignant tumor of the digestive tract; rectal adenocarcinoma (READ) is a subtype that accounts for about 20%–30% of the total cases (Flam et al., 2022). The clinical outcome and quality of life of these patients remain suboptimal (Emile et al., 2023). Therefore, identifying molecular markers and constructing accurate prediction models to optimize treatment strategies and improve patient prognosis holds clinical significance.

In 2022, “cuproptosis” was first described as a new cell death mechanism (Tsvetkov et al., 2022). Studies have shown that this process is closely associated with the tricarboxylic acid cycle (TCA) (Oliveri, 2022). Copper binds directly to the fatty part of the TCA cycle, and the production of iron-sulfur cluster proteins is downregulated, leading to the accumulation of fatty acylated proteins, producing proteotoxic stress and ultimately resulting in cell death (Tsvetkov et al., 2022).

Notably, tumor metabolic reprogramming phenomena (such as the Warburg effect, abnormal fatty acid metabolism, etc.) not only provide energy for cancer cell proliferation (Boroughs and DeBerardinis, 2015; Tang et al., 2022). In addition, its metabolites can also affect the function of immune cells by remodeling the tumor microenvironment (TME) and enhancing the ability of tumor immune escape (Altman et al., 2016). Therefore, enhancing the immune escape of tumor cells may affect the treatment outcome. Growing evidence supports the role of the TME in the occurrence and progression of READ, thereby affecting the therapeutic outcomes (Ciardiello et al., 2022; Zhang et al., 2023). Recent studies have shown that nano-drug delivery systems based on cuproptosis can exert an anti-tumor effect by regulating the TME (Li et al., 2024). This provides a new theoretical basis for exploring the immunotherapy strategy of READ.

Compared with colon adenocarcinoma (COAD), READ has significant differences in embryonic development origin, clinical treatment pathway, and gene expression characteristics (Paschke et al., 2018; Tamas et al., 2015). Previous studies have systematically analyzed cuproptosis-related genes (CRGs) in COAD (Gu et al., 2024); in contrast, no multi-omics studies investigating CRGs in READ have been performed. This study is the first to construct a CRG prognostic prediction model for READ by integrating genomics, transcriptomics, and clinical data. The reliability of the model was verified through multiple dimensions, such as risk stratification survival analysis, immune infiltration profile analysis, and drug sensitivity evaluation.

## Methods

### Multi-omics data sources and processing

In this study, the transcriptome and clinical data of READ patients were downloaded from The Cancer Genome Atlas (TCGA) (https://www.cancer.gov/, accessed on January 2, 2025) and Gene Expression Omnibus (GEO) https://www.ncbi.nlm.nih.gov/geo/, accessed on January 2, 2025) databases. The TCGA-READ dataset comprised 171 samples (including tumor and normal tissues). The raw data were stored in the Genomic Profiling (FPKM) format and normalized to the transcripts per million (TPM) format for compatibility and comparison with microarray data. The GEO dataset GSE87211 was downloaded. The cuproptosis genes were identified through relevant literature (Chen et al., 2024) and the FerrDbV2 database (http://www.zhounan.org/ferrdb/current/operations/download.html). The GEO dataset GSE87211 (including 203 tumor and 160 normal samples) was integrated with the TCGA-READ data (including 167 tumor and 10 normal samples) for the differential expression analysis of CRGs and the identification of molecular subtypes.

### Multi-dimensional feature analysis of CRGs

The CRG mutation spectrum and tumor mutation burden (TMB) were visually analyzed using the “maftools” toolkit. After integrating the TCGA and GEO data, the survival/survminer package was utilized to carry out univariate Cox regression to evaluate the prognostic value. Finally, the CRG interaction network was constructed based on Pearson correlation.

### Cuproptosis subtypes and immune microenvironment characteristics

Unsupervised clustering based on the CRG expression profile was performed using the “ConsensusClusterPlus” package to identify READ subtypes, and subtype stability was verified by Principal Components Analysis (PCA) dimensionality reduction. The differences in survival across subtypes were compared using the Kaplan-Meier curve, while the chi-square test was employed to examine the relationship between clinical characteristics. Gene Set Variation Analysis (GSVA) (Hanzelmann et al., 2013) and single sample Gene Set Enrichment Analysis (ssGSEA) (Charoentong et al., 2017) were used to explore the heterogeneity of KEGG pathways and 23 types of immune cell infiltration characteristics between groups, respectively.

### Screening and functional annotation of differential genes

The differential threshold (|logFC| ≥0.585, adj. p < 0.05) was set to identify DEGs among subtypes, and GO/KEGG enrichment analyses were conducted to reveal the biological processes related to cuproptosis. A Venn diagram was used to visualize the intersection of core genes and highlight key regulatory nodes (Yu et al., 2012).

### Construction and validation of the CRG prognostic model

This investigation adopted a prospective cohort design, and 177 cases were randomly assigned to model development and validation cohorts. LASSO-Cox regression analysis was performed, and the optimal combination of cuproptosis-related genes was identified to establish a quantitative prognostic scoring system. To validate the prognostic model, patients were divided based on median risk thresholds. The predictive performance was assessed using time-dependent ROC curves and Kaplan-Meier survival differential analyses to compare survival differences between the low-risk and high-risk groups. Dynamic Sankey mapping was used to visualize the relationships between risk stratification and clinicopathological parameters. Nonparametric statistical tests were employed to evaluate the discriminative capacity of the scoring system across clinical subgroups. Multivariate regression models indicated that the risk score was an independent prognostic indicator, with subgroup stratification analyses demonstrating consistent predictive performance across age, gender, and TNM stage categories. A clinically applicable nomogram integrating conventional parameters and risk scores demonstrated superior predictive capability, as evidenced by calibration curves showing strong concordance between predicted and observed 1-/3-/5-year survival probabilities.

### Immune landscape multi-algorithm analysis

The ESTIMATE algorithm (Yoshihara et al., 2013) was used to quantify tumor purity and stroma/immune score, and the CIBERSORT algorithm was used to analyze the infiltration proportion of 22 immune cells (Newman et al., 2019). Six algorithms, including TIMER2.0, quanTlseq, and xCell, were used to cross-validate the characteristics of immune microenvironment to reveal the differences in immune suppression between high and low risk groups (Li et al., 2017; Finotello et al., 2019; Aran et al., 2017; Becht et al., 2016; Racle et al., 2017).

### Analysis of drug sensitivity and immunotherapy

To evaluate the potential clinical utility of the cuproptosis risk model in predicting treatment response, we analyzed sensitivity to a curated list of chemotherapeutic and targeted agents. The drug selection was hypothesis-driven, focusing on two categories: (1) chemotherapeutic drugs commonly used in the standard-of-care for colorectal cancer; and (2) targeted agents with known links to metabolic pathways or potential relevance to cuproptosis-related mechanisms. The relationship between the expression levels of 47 immune checkpoint genes and CRGS risk scores was examined, utilizing the immunophenotypic score (IPS) from the TCIA database (https://tcia.at/home) to identify patients likely to benefit from immunotherapy. The IC50 values for chemotherapeutic agents were computed using the pRRophetic package to identify drugs (Geeleher et al., 2014).

### Statistical analysis

All analyses were performed based on R 4.4.2 with a significance threshold of p < 0.05. The Bootstrap method was used for internal verification to ensure the reproducibility of the results.

## Results

### Difference analysis of cuproptosis-related genes

The TCGA-READ dataset includes a total of 177 samples from READ patients and normal tissues.

**FIGURE 1:**
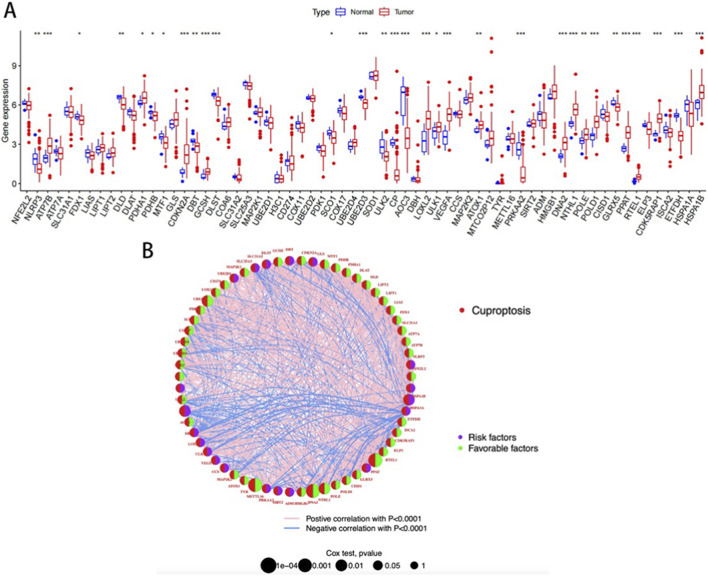
**(A)** Differential expression of CRGs; **(B)** Correlation network diagram of CRGs.

The TCGA database analysis of the expression of 65 cuproptosis-related genes revealed that compared with normal patients, 16 genes (NLRP3, PDHA1, CDKN2A, GCSH, LOXL2, ULK1, VEGFA, ATOX1, DNA2, NTHL1, POLE, POLD1, PPRAT, RTEL1, CDK5RAP1, HSPA1B) were upregulated in READ patients. Conversely, 14 genes (ATP7B, FDX1, DLD, PDHB, MTF1, DBT, SCO1, DLST, UBE2D3, ULK2CP, AOC3, PRKAA2, GLRX5, and ETFDH) were downregulated. Thereafter, a network diagram depicting the interactions among the 65 CRGs was developed (Figure 1).

A blue line indicates a negative correlation between two genes, while a pink line indicates a positive correlation. Line thickness indicates the strength of the correlation. The size of the circles indicates the prognostic significance of CRG, with p values classified as p < 0.0001, p < 0.001, p < 0.01, p < 0.05, and p < 1. The purple semi-circle and the green semi-circle represent the risk factor and the protective factor, respectively.

### Functional characterization of cuproptosis-related genes in READ tumor microenvironment based on molecular subtyping

**FIGURE 2:**
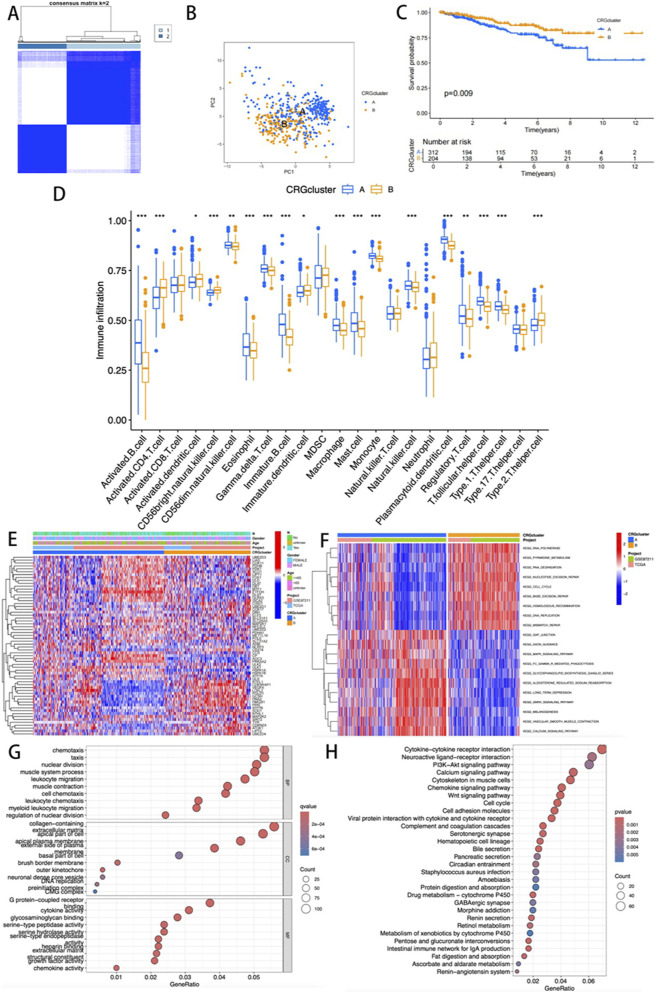
**(A)** Consensus matrix map of two relevant regions; **(B)** PCA showing the differences between the two subtypes; **(C)** K-M survival analysis curve; **(D)** Evaluation of differences in immune infiltration among 23 key immune cells; **(E)** The heatmap visualization illustrates the differences in clinical pathological parameters among various CRG expression profile subtypes; **(F)** GSVA mapping of pathway activities across different clusters. **(G,H)** GO and KEGG functional enrichment analysis.

In this study, systematic consensus clustering was performed on 65 CRGs using cluster parameter k ranging from 2 to 9. At k = 2, the cohort was distinctly divided into two molecular subtypes with divergent biological features: cuproptosis cluster A (n = 319) and cluster B (n = 210) (Figure 2A). PCA confirmed significant molecular heterogeneity between the subtypes (Figure 2B). Survival analysis demonstrated that cluster A patients exhibited markedly prolonged overall survival (OS) compared to cluster B (p = 0.009, Figure 2C), highlighting the prognostic significance of this subtyping framework.

Quantitative analysis of immune infiltration revealed enhanced activation of immune cells in cluster A. Among 21 immune cell subsets, 18 showed statistically significant differences (p < 0.05), with CD8^+^ T cells, MDSCs, NKT cells, and Th17 cells predominantly enriched in cluster A, whereas higher levels of activated CD4^+^ T cells, dendritic cells, NK cells, and Th2 cells were observed in cluster B (Figure 2D). Heatmap visualization of clinicopathological correlations indicated comparable distributions of age, sex, and N-stage between the two clusters (Figure 2E).

GSVA revealed distinct functional enrichment patterns. Cluster B was predominantly associated with nucleic acid metabolism pathways (e.g., pyrimidine metabolism, RNA synthesis, and base excision repair; Figure 2F), whereas cluster A showed activation of MAPK signaling, gonadotropin-releasing hormone (GnRH) signaling, and calcium ion transport pathways. Differential expression analysis identified 1,962 differentially expressed genes (DEGs), offering critical insights into the molecular mechanisms underlying cuproptosis.

GO and KEGG pathway analyses of DEGs highlighted their enrichment in immune microenvironment-related processes, including chemokine regulation and collagen-containing extracellular matrix remodeling (Figure 2G). Furthermore, KEGG analysis revealed that these genes were involved in cytokine-cytokine receptor interactions, neuroactive ligand-receptor crosstalk, and PI3K-Akt signaling (Figure 2H). Collectively, these multi-omics findings suggest that CRGs may drive READ progression by regulating immunomodulatory networks and metabolic reprogramming within the tumor microenvironment.

### Molecular typing was conducted based on DEGs

**FIGURE 3:**
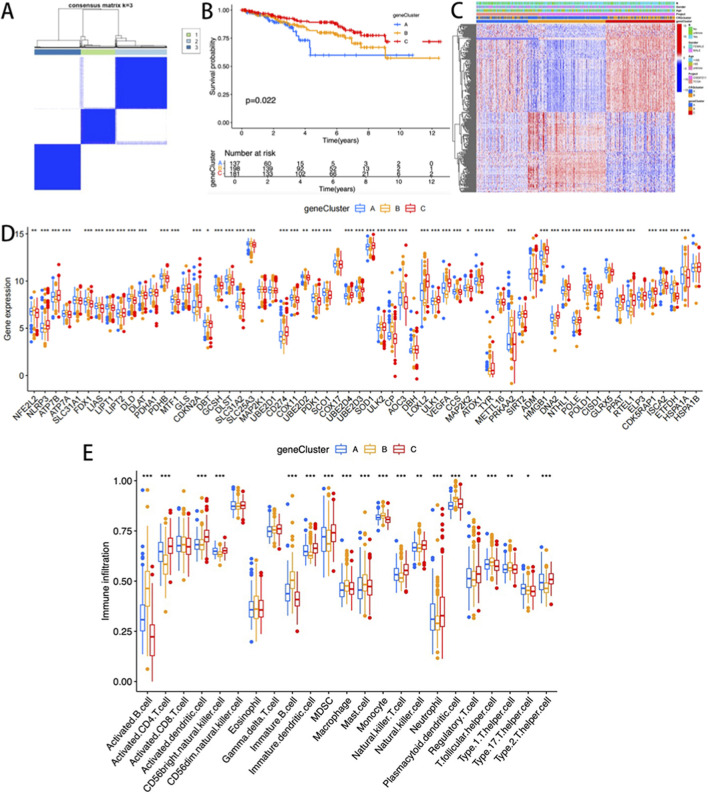
**(A)** Consensus clustering matrix (k = 3); **(B)** K-M curves of the three gene subtypes; **(C)** The heatmap visualization illustrates the differences in clinical pathological parameters among three gene clusters; The differences in the expression level; **(D)** and immune cell infiltration level; **(E)** of CRGs among the three gene clusters.

Univariate Cox regression analysis identified 1,962 subtype-related genes, and 530 prognosis-related genes were retained for molecular characterization and survival-related studies. The consistent clustering algorithm was employed to stratify patients based on these prognostic markers, generating three distinct molecular subgroups (optimal k = 3): Group A (n = 139), Group B (n = 207), and Group C (n = 185) (Figure 3A). The stratified analysis of overall survival rate (OS) demonstrated that the survival rate of Group A was significantly lower compared to Groups B and C (p < 0.001, Figure 3B). No significant differences were observed among the three clusters in terms of clinicopathological parameters (Figure 3C). Among the three molecular subgroups, the expression levels of CRGs exhibited significant heterogeneity (Figure 3D). Further analysis determined the specific immune infiltration patterns in the three gene subtypes, and Gene cluster C showed an increase in immunocompetent cells, including activated CD4T cells, activated dendritic cells, activated CD56NK cells, immune dendritic cells, MDSC, NKT cells, Neutrophil, regulatory T cells, and type 2 T cell helper cells (Figure 3E).

### Development and validation of a cuproptosis-related prognostic model

**FIGURE 4:**
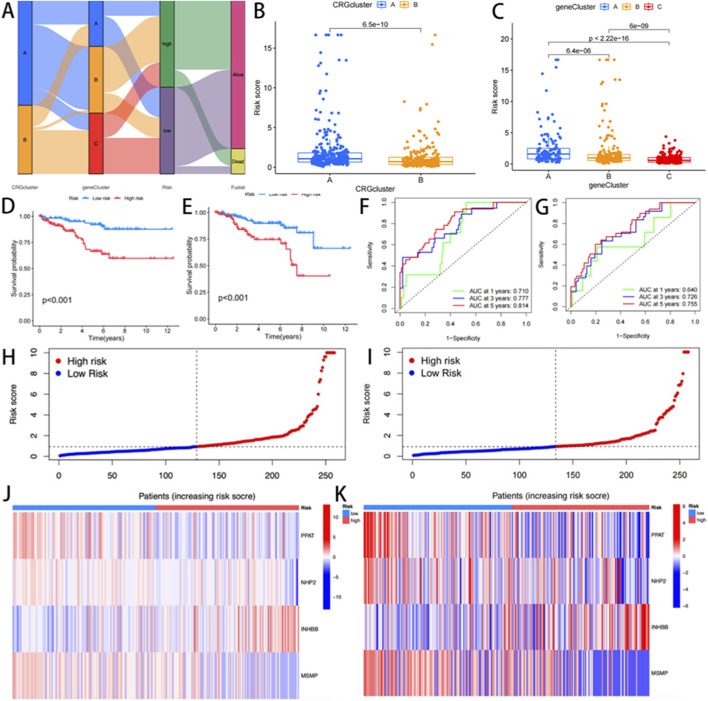
**(A)** Sankey Diagram; **(B,C)** Differences in cuproptosis risk scores; **(D,E)** Differential analysis of gene clusters related to cuproptosis K-m survival analysis between the training group and the experimental group; **(F,G)** ROC curves for predicting 1 -, 3 -, and 5-year survival; **(H,I)** risk distribution maps; **(J,K)** genotyping heat maps of the high-risk and low-risk patient groups.

A risk stratification model was constructed based on DEGs associated with cuproptosis subtypes. The integrative analysis revealed associations between molecular clusters, risk scores, survival outcomes, and cuproptosis classification (Figure 4A). Subsequently, the cohorts were divided into the training (n = 250) and validation (n = 249) sets at a 1:1 ratio. LASSO-Cox regression analysis of cuproptosis-associated DEGs identified four prognostic genes, including PPAT and NHP2 as protective factors, and INHBB and MSMP as risk factors. Stratification based on median risk scores categorized 277 CRGs as low-risk and 220 as high-risk. Elevated risk scores correlated with reduced survival duration and increased mortality (Figure 4D,E). Significant inter-cluster heterogeneity was observed. Notably, when we examined two cuproptsis clusters using three gene clusters, we found that the risk rating of cluster A was much higher (p < 0.001, Figure 4B). Meanwhile, gene cluster A exhibited a significantly higher CRGs risk score, and all three clusters showed statistically significant differences (p < 0.001, Figure 4C).

Furthermore, Kaplan-Meier analysis demonstrated markedly prolonged OS in low-risk patients compared to high-risk patients (p < 0.001; Figure 4H,I). The prognostic model showed robust discriminative capacity, with time-dependent AUC values of 0.710 (1-year), 0.777 (3-year), and 0.814 (5-year) in the training cohort (Figure 4F), and 0.640, 0.726, and 0.755 in the validation cohort, respectively (Figure 4G). A genotype-expression heatmap further confirmed the model’s predictive reliability across both cohorts (Figure 4J,K).

### Prognostic model construction and clinical validation

**FIGURE 5:**
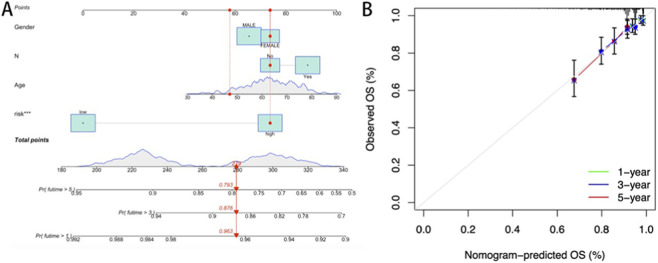
**(A)** Nomogram constructed based on the risk score and clinicopathological characteristics; **(B)** Calibration curve of the prognostic model.

To validate the prognostic utility of the cuproptosis risk signature in stratifying READ patient outcomes, a multifactorial prognostic model integrating the risk score with clinicopathological variables was constructed (Figure 5A). Calibration plots demonstrated strong concordance between the predicted and observed OS probabilities at 1-, 3-, and 5-year intervals (Figure 5B), validating the discriminative accuracy of this nomogram for individualized survival estimation.

### Immune profiling stratified by cuproptosis risk stratification

**FIGURE 6:**
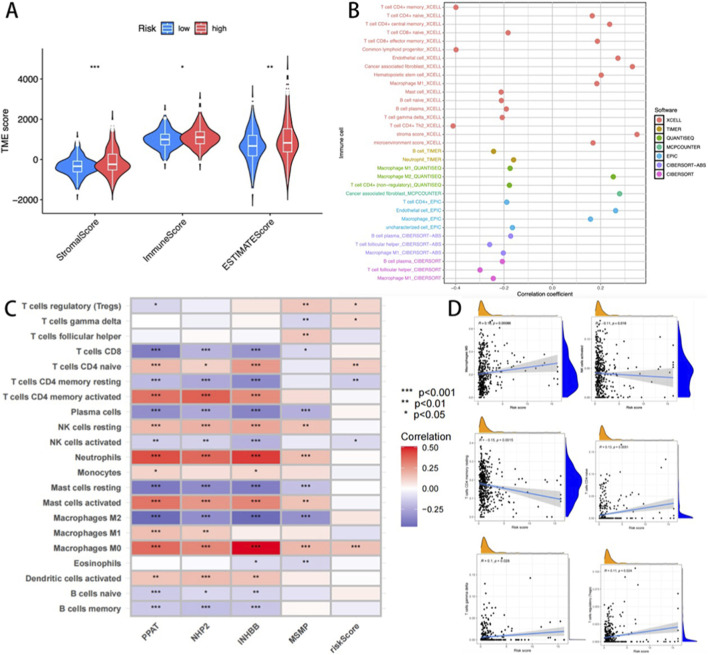
**(A)** The differences between the high-risk and low-risk groups across the three immune algorithms; **(B)** Heat maps describing the changes in the content of immune cells in different algorithms; **(C)** Heat map of the correlation between immune cells and four key genes. **(D)** Correlation between cuproptosis risk score and six key immune cells (M0 Macrophages, activated NK cells, CD4 memory resting T-cells, CD4 naïve T-cells, gamma delta T-cells, and regulatory T-cells (Tregs).

Considering the pivotal effects of the tumor microenvironment (TME) on oncological outcomes, we conducted an immune correlation analysis based on the risk stratification related to CRGs. TME scoring via the ESTIMATE algorithm revealed elevated stromal, immune, and composite scores in high-risk patients compared to low-risk counterparts (p < 0.001; Figure 6A). Multi-algorithmic deconvolution (TIMER, CIBERSORT, quanTIseq, etc.) quantified immune infiltration heterogeneity, visualized through a functional annotation heatmap (Figure 6B).

Prognostic gene-immunome correlation analysis identified a positive association between INHBB and macrophage and neutrophil infiltration (Figure 6C). The cuproptosis risk score demonstrated significant covariation with six immunophenotypic subsets, including M0 Macrophages, activated NK cells, CD4 memory resting T-cells, CD4 naïve T-cells, gamma delta T-cells, and regulatory T-cells (Tregs) (Figure 6D). These findings highlight the model’s capacity to reflect TME immunoreactivity, suggesting its utility in guiding READ patient immunotherapy stratification.

### Therapeutic response prediction and biological relevance of the cuproptosis risk model

**FIGURE 7:**
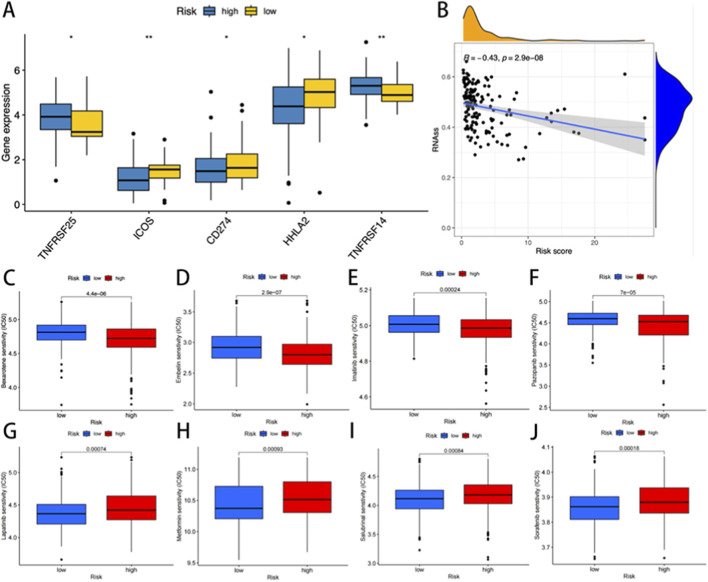
**(A)** The expression of immune checkpoint genes in the high and low-risk groups. **(B)** Correlation analysis between cuproptosisrisk score and stem cells. The IC50 was significantly reduced for drugs such as high-risk bexarotene, embelin, imatinib, and pazopanib **(C–F)**. In contrast, the low-risk group showed significantly lower IC50 levels for lapatinib, metformin, salubrinal, and sorafenib **(G–J)**.

To evaluate the predictive capacity of the cuproptosis risk model for therapeutic response, immune checkpoint (ICP) expression profiles were first analyzed across risk-stratified cohorts. Among 47 evaluated ICP genes, five were differentially expressed between the two risk groups (p < 0.05, Figure 7A). Notably, low-risk patients demonstrated elevated expression of ICOS, CD274 (PD-L1), and HHLA2 (p < 0.01, p < 0.05, and p < 0.05, respectively), while showing reduced levels of TNFRSF25 and TNFRSF14 compared to the high-risk patients (p < 0.05 and p < 0.01). Drug sensitivity analysis of the GDSC pharmacogenomic data revealed that high-risk patients exhibited increased sensitivity to irinotecan (p < 0.001), a topoisomerase I inhibitor commonly employed in READ treatment (Figure 7C–J). Notably, an inverse correlation was observed between cuproptosis risk scores and tumor stemness indices (p < 0.001, Figure 7B). These findings suggested that high-risk tumors may display reduced cellular plasticity and differentiation potential.

## Discussion

In this study, a multi-dimensional prognostic analysis system was established by evaluating molecular subtype, tumor heterogeneity, and immune microenvironment. The impact of CRGs on the survival of READ patients was assessed by constructing a CRGs-related prognostic model, and the stability of the model was enhanced by internal and external validation. In addition, CRGs were analyzed in combination with drug sensitivity data to predict the effectiveness of drugs, facilitating the development of personalized treatment strategies.

As a common subtype of colorectal cancer, READ has significant differences in biological characteristics, treatment, and prognosis. The copper-dependent cell death mechanism has attracted growing attention as a therapeutic target. Previous studies have revealed that CRGs are associated with prognosis in a variety of tumors (Bian et al., 2022; He et al., 2024); however, the mechanism of their role in READ remains unknown.

In this study, the expression of 65 cuproptosis-related genes was analyzed based on the TCGA database. Next, two subgenotypes showing significant survival differences were identified based on the differentially expressed CRGs, revealing the potential value of CRGs in predicting immunotherapy response. Additionally, a mortality risk score system was constructed based on the INHBB, PPAT, NHP2, and MSMP genes. Finally, its prognostic efficacy was verified.

As a core regulator of glutamine metabolism, PPAT (Phosphoribosyl pyrophosphate Amido transferase) plays an important role in tumor development by mediating the purine nucleotide biosynthesis pathway (Hanahan and Weinberg, 2011). Studies have shown that the expression of PPAT is directly regulated by the proto-oncogene c-Myc, and its abnormal high expression can promote the rapid proliferation of cancer cells by accelerating the production of purine nucleotides (Kodama et al., 2020). Clinical data analysis revealed a significantly higher transcriptional level of PPAT in colorectal cancer tissues, and its expression intensity was found to be negatively correlated with the survival rate of patients (Khalili-Tanha et al., 2023). In the TGF-β signaling pathway system, the protein molecules encoded by the activin Bβ subunit (INHBB) gene have a wide range of biological functions. Its products mediate Smad-dependent and independent signal transduction through activin receptor kinase, playing a key regulatory role in the process of tumor cell migration and invasion (Yuan et al., 2020; Goney et al., 2020; Liu et al., 2024). Notably, INHBB overexpression significantly enhanced tumor metastasis by inducing epithelial-mesenchymal transition (EMT). Preclinical studies confirmed that knockdown of this gene effectively inhibited tumor proliferation, suggesting its potential as a prognostic marker for high-risk COAD (Yuan et al., 2020). Telomerase is a polymeric ribonucleoprotein complex that catalyzes the elongation of telomeric repeats at chromosomal termini and demonstrates functional deficiencies closely associated with progressive telomere shortening during somatic cell senescence. Notably, dysregulated telomere attrition or compromised telomeric integrity has emerged as a therapeutic target in oncology (Guterres and Villanueva, 2020). Mechanistically, NHP2 functions as a core functional subunit of this complex; dysregulated expression of NHP2 is significantly correlated with malignant progression in colorectal malignancies (Shay and Wright, 2019; Tao et al., 2023). Experimental evidence demonstrated age-associated NHP2 overexpression in CRC pathological specimens, while subsequent *in vitro* and *in vivo* models confirmed its pivotal role in tumor progression through telomere homeostasis modulation, thereby mediating critical oncogenic biological processes (Gong et al., 2021). Microprotamine (MSMP) is a highly conserved secreted protein containing signal peptide sequences. Although its molecular structure lacks typical chemokine characteristics, it is specifically highly expressed in hormone-resistant prostate cancer cells. Recent studies have revealed that MSMP regulates monocyte infiltration by interacting with the CCR2 receptor and may play a role in remodeling the TME; nonetheless, the specific molecular mechanism remains poorly understood (Mitamura et al., 2018). Although our prognostic model has established the key value of these four genes in READ, it must be admitted that there are still relatively few literature reports on the direct molecular mechanisms of PPAT, NHP2, INHBB and MSMP in cuproptosis. In particular, the roles of INHBB and MSMP in READ remain unexplored, which provides an exciting approach for future research. Therefore, one of the significant meanings of this study lies in that it has, for the first time, revealed through multi-omics analysis the strong association between this group of genes and the prognosis of READ, thereby providing a brand-new and clear target for subsequent mechanism research. We speculate that these genes may not be the core executors of cuproptosis, but rather function as important regulatory nodes or effector molecules, linking the metabolic crisis induced by cuproptosis to broader tumor biological behaviors.

Cuproptosis, as a newly discovered form of cell metabolism-related cell death, has complex interactions with the TME. The tumor microenvironment is mainly composed of immune cells and stromal cells, and the degree of cell infiltration has a significant impact on the prognosis of patients (Basak et al., 2023). Studies have shown that CRGs are significantly enriched in metabolic pathways and can promote tumor progression by influencing the TME (Gu et al., 2024). In terms of mechanism, studies have shown that copper death can cause tumor cells to release damage-related molecular patterns (DAMPs), such as mitochondrial DNA, which in turn activate the cGAS-STING signaling pathway and promote the secretion of inflammatory factors such as type I interferons (IFNB) and chemokines (such as CXCL10). Recruit immune cells such as dendritic cells (DC), CD8^+^ T cells and NK cells to enhance the anti-tumor immune response (Guo et al., 2025). Therefore, cuproptosis may play an important immunomodulatory role in the TME by regulating the infiltration and activation of immune cells. In this study, risk stratification analysis revealed significant differences in stromal activity, immune features, and composite scores between the high- and low-risk groups. Tumor purity was negatively correlated with risk scores but positively linked to stromal and immune markers. High risk scores may drive tumor progression, worsening clinical outcomes. The survival data revealed shorter overall survival in high-risk patients, indicating potential connections between rectal cancer prognosis and immune cell infiltration levels. Immune cell quantification and tumor growth patterns offer new insights for refining prognostic models. Furthermore, immune checkpoint molecules not only act as biomarkers of treatment response but also play a crucial role in the process of tumor immune escape (Randrian et al., 2021). The development of immune checkpoint blockade therapy (ICB) targeting such molecules provides a new therapeutic direction for patients with related cancers (Weng et al., 2022). The efficacy of ICB therapy is regulated by multiple factors, including the degree of tumor immune infiltration, checkpoint expression level, tumor mutation burden, and neoantigen formatio (Marisa et al., 2018). The present study indicated that the cuproptosis risk score was positively correlated with the expression levels of the three key immune checkpoint targets. Gene expression levels in the high-risk cuproptosis group were significantly higher than those in the low-risk group. Tumor cells within this cohort might evade the immune response by activating checkpoint molecules. Meanwhile, according to the analysis of tumor immune correlation, the infiltration of M0 macrophages and regulatory T cells is higher in the high-risk group, which is usually related to tumor progression and immune evasion. These findings have significant clinical implications for immunotherapy. The unique immune background of high-risk populations, particularly inhibitory cellular components, suggests that these patients may exhibit inherent resistance to immune checkpoint blockade therapy. This is consistent with the differential expression of immune checkpoint genes (such as CD274) observed between risk groups. The differences in drug sensitivity of patients from different risk groups were compared. Lapatinib, metformin, salubrinal, and sorafenib showed lower sensitivity in low-risk patients, whereas bexarotene, embelin, imatinib, and pazopanib demonstrated lower sensitivity in high-risk patients. The drug resistance characteristics of READ patients can be predicted before chemotherapy by using the cuproptosis risk score, providing a molecular basis for the development of individualized treatment plans and optimizing clinical medication strategies.

It is important to note that our study has certain limitations. Firstly, this study lacks validation from a large independent prospective cohort. Despite rigorous internal validation, future research utilizing external multicenter cohorts is crucial for further validating the universality and clinical applicability of our cuproptosis-related prognostic model. Future work will focus on collecting clinical samples to validate the protein expression of these key genes via immunohistochemistry or ELISA, and on establishing multi-center collaborations to prospectively validate the model’s performance. In addition, although the clinical value of CRGs has been systematically analyzed by bioinformatics methods, prospective clinical studies are still required to verify the predictive efficacy of the model. At present, the proposed drug sensitivity conclusions are primarily based on computational models; however, their clinical applicability must be comprehensively evaluated to determine their pharmacokinetics and toxic side effects. Future studies should focus on constructing the regulatory network of CRGs and exploring the interaction mechanism between CRGs and epigenetic modification, providing theoretical support for the development of novel READ therapeutic drugs.

## Conclusion

The CRG scoring system has the potential to predict prognosis with great accuracy and introduces new avenues for cuproptosis as a therapeutic target in the treatment of READ. A deeper understanding of the molecular pathways involved in READ will facilitate the development of more targeted treatment alternatives.

## Data Availability

The original contributions presented in the study are included in the article/supplementary material, further inquiries can be directed to the corresponding author.
